# Shrinking lung syndrome in systemic lupus erythematosus: A case series and review of the literature

**DOI:** 10.1097/MD.0000000000004626

**Published:** 2016-08-19

**Authors:** Helena Borrell, Javier Narváez, Juan José Alegre, Ivan Castellví, Francesca Mitjavila, María Aparicio, Eulàlia Armengol, María Molina-Molina, Joan M. Nolla

**Affiliations:** a Department of Rheumatology, Hospital Universitario de Bellvitge-IDIBELL, Barcelona; b Department of Rheumatology, Hospital Universitario Dr. Peset, Valencia; c Rheumatology Unit, Hospital de Sant Pau; d Department of Internal Medicine; e Department of Pneumology, Hospital Universitario de Bellvitge-IDIBELL, Barcelona, Spain.

**Keywords:** imaging techniques, physiopathology, shrinking lung syndrome, systemic lupus erythematosus

## Abstract

Shrinking lung syndrome (SLS) is a rare and less known complication mainly associated with systemic lupus erythematosus (SLE). In this study, we analyze the clinical features, investigation findings, approaches to management, and outcome in a case series of 9 adult patients with SLE and SLS diagnosed during a 35-year period in 3 referral tertiary care hospitals in Spain. Additionally, we reviewed 80 additional cases previously reported (PubMed 1965–2015). These 80 cases, together with our 9 patients, form the basis of the present analysis.

The overall SLS prevalence in our SLE population was 1.1% (9/829). SLS may complicate SLE at any time over its course, and it usually occurs in patients without previous or concomitant major organ involvement. More than half of the patients had inactive lupus according to SELENA-systemic lupus erythematosus disease activity index (SLEDAI) scores. Typically, it presents with progressive exertional dyspnea of variable severity, accompanied by pleuritic chest pain in 76% of the cases.

An important diagnostic delay is common. The diagnostic tools that showed better yield for SLS detection are the imaging techniques (chest x-ray and high-resolution computed tomography) along with pulmonary and diaphragmatic function tests. Evaluation of diaphragm dome motion by M-mode ultrasonography and phrenic nerve conduction studies are less useful.

There are no standardized guidelines for the treatment of SLS in SLE. The majority of patients were treated with medium or high doses of glucocorticoids. Several immunosuppressive agents have been used in conjunction with steroids either if the patient fails to improve or since the beginning of the treatment. Theophylline and beta-agonists, alone or in combination with glucocorticoids, have been suggested with the intent to increase diaphragmatic strength.

The overall long-term prognosis was good. The great majority of patients had significant clinical improvement and stabilization, or mild to moderate improvement on pulmonary function tests. The mortality rate was very low.

## 1 Introduction

Shrinking lung syndrome (SLS) is a rare and less known complication mainly associated with systemic lupus erythematosus (SLE), although it has also been reported in patients with other connective tissue diseases (CTDs), including primary Sjögren syndrome, scleroderma, rheumatoid arthritis, and undifferentiated connective tissue disorder.^[1–4]^ It is characterized by progressive dyspnea, usually accompanied by pleuritic chest pain, diaphragmatic elevation, lung volume reduction with no parenchymal abnormalities, and a restrictive ventilatory defect in pulmonary function tests (PFTs).

Many aspects of the disorder remain to be elucidated: its exact prevalence is unknown, the underlying pathophysiology is unclear, and the best treatment and prognosis remain controversial.

In the present study, we analyze the clinical features, investigation findings, approaches to management, and outcomes in a series of adult patients with SLS associated with SLE. We also reviewed the available literature to summarize the experience with this complication.

## 2 Patient and methods

### 2.1 Patient selection

The sample included all adult patients (n = 9) with SLS and SLE (all of whom met the American College of Rheumatology classification criteria for SLE),^[5]^ who were routinely treated from January 1980 to May 2015 at the rheumatology departments of 3 referral tertiary care hospitals in Spain and followed for at least 6 months after SLS diagnosis. A retrospective analysis of prospectively collected data was performed.

Patients were considered to have an SLS if the following conditions were present: a compatible clinical picture (progressive exertional dyspnea of variable severity with or without pleuritic chest pain); lung volume reduction and restrictive ventilatory defect in PFT; and no evidence of parenchymal lung disease or vascular pathology on imaging (chest x-ray and thoracic high-resolution computed tomography [HRCT]) findings. The inpatient and outpatient charts of the patients were comprehensively reviewed by the study investigators to obtain clinical, laboratory, and disease course data.

The medical records of the patients with SLS and SLE were transcribed onto a specific form that included the following information: baseline demographic characteristics; disease duration; other previous and concomitant clinical features of lupus; SELENA-systemic lupus erythematosus disease activity index (SLEDAI) at SLS diagnosis; presenting features of SLS; diagnostic studies; treatment; and long-term outcome.

In accordance with the guidelines of our institutional ethics committee, formal approval for this study was not required. The local ethics committee agreed that the findings in this report were based on normal clinical practice and were therefore suitable for dissemination. Informed consent was not obtained from the patients, but their clinical records and information were anonymized before analysis. This study was conducted in accordance with the principles of the Declaration of Helsinki and the International Conference for Harmonization.

### 2.2 Literature search strategy and selection criteria

Searches were conducted in the PubMed database (i.e., including MEDLINE, National Library of Medicine, and PubMed Central) for the period between January 1965 and November 2015, using strategies recommended by the Cochrane handbook. The keyword used was “shrinking lung syndrome.” Only English, French, Portuguese, and Spanish reports were considered. To standardize the information, we excluded: patients aged less than 18 years, pregnant women, and patients with overlap syndromes or with SLS associated with other CTDs different from SLE.

Reference lists of original studies were manually searched, and relevant articles were extracted. The references of the studies obtained were also examined to identify additional reports. The manuscripts of all potentially relevant studies identified during the search of abstracts were then retrieved and reviewed.

**Figure 1:**
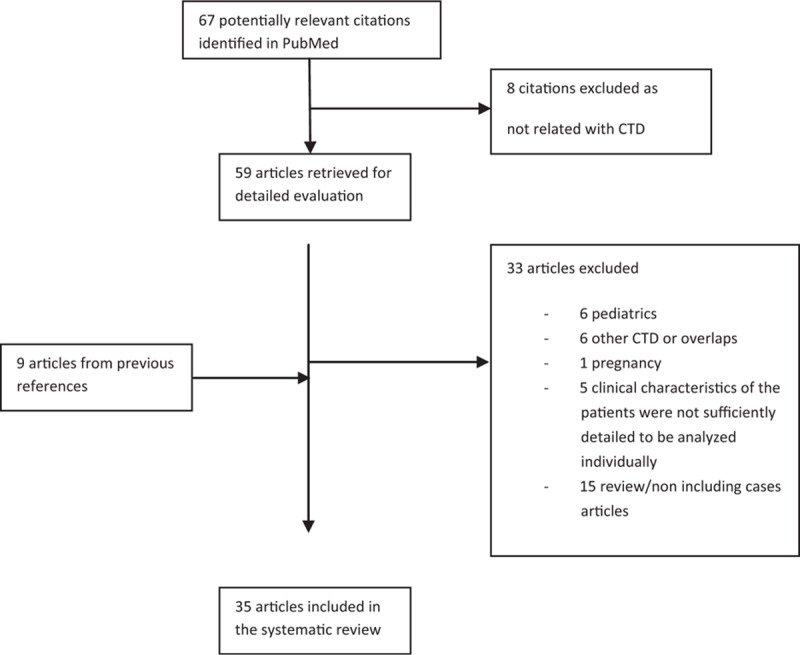
MEDLINE search: process selection.

The MEDLINE search resulted in 67 articles (Fig. 1). Of them, 8 were excluded because they were not related with CTD.^[6–13]^ The remaining 59 articles were evaluated, together with 9 additional reports identified from the revision of the references.

After evaluation of the full text, 33 of these articles were excluded: 15 were a review or contained irrelevant data^[14–28]^; 7 corresponded to pregnant or pediatric cases^[29–35]^; and 6 were overlap syndromes^[36,37]^ or cases of SLS associated with other CTDs different from SLE.^[1–4]^ Finally, we also excluded the cases reported by Allen et al^[38]^ (11 cases), Gibson et al^[39]^ (7 cases), Gheita et al^[40]^ (8 cases), Elkayam et al^[41]^ (2 cases), and 1 case published by Rubin and Urowitz^[42]^ because their clinical characteristics were not sufficiently detailed to be individually analyzed.

Therefore, 35 articles were finally selected for review, identifying 80 well-documented cases of adult SLE patients with SLS.^[43–77]^

### 2.3 Statistical analysis

Qualitative variables were described by frequencies and percentages, and quantitative variables by mean or median ± standard deviation (SD) and range.

## 3 Results

The main clinical characteristics and outcome of the 9 patients with SLS and SLE diagnosed in our institutions are summarized in Table 1. The overall SLS prevalence in our lupus population during a 35-year period was 1.1% (9/829).

**Table 1:**
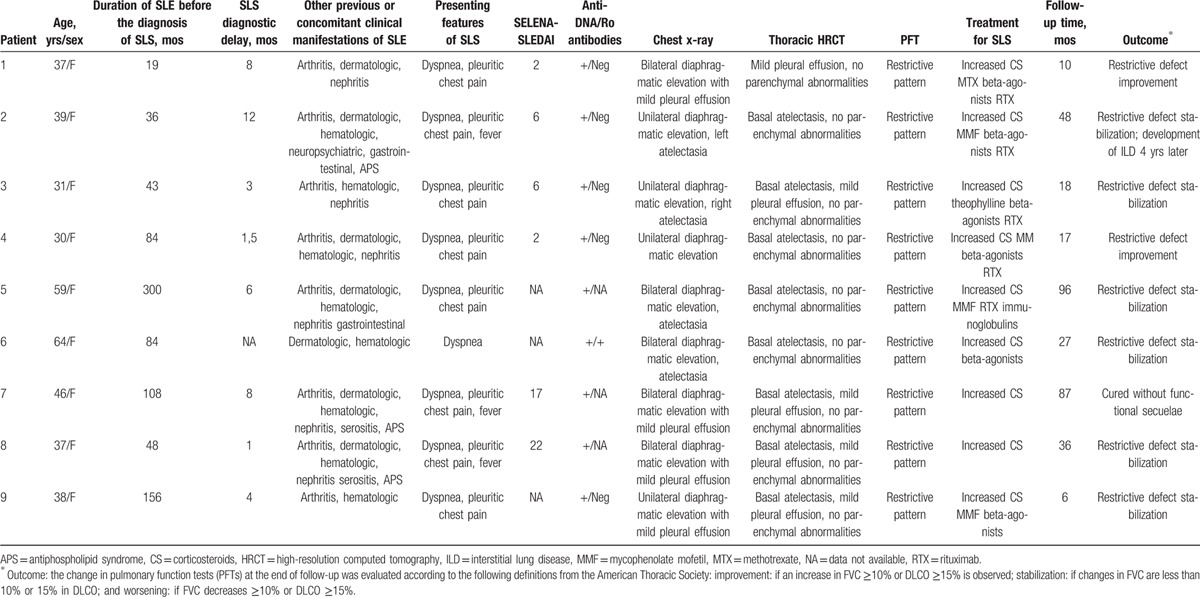
Main clinical characteristics and outcome of our patients.

The main clinical characteristics and outcome of the 80 patients obtained from the literature are summarized in Table 2. Because it is a review of the literature, not all analyzed variables were recorded in all published cases. Thus, the results (percentages) in each variable were calculated considering only the number of patients in which the data have been documented.

**Table 2:**
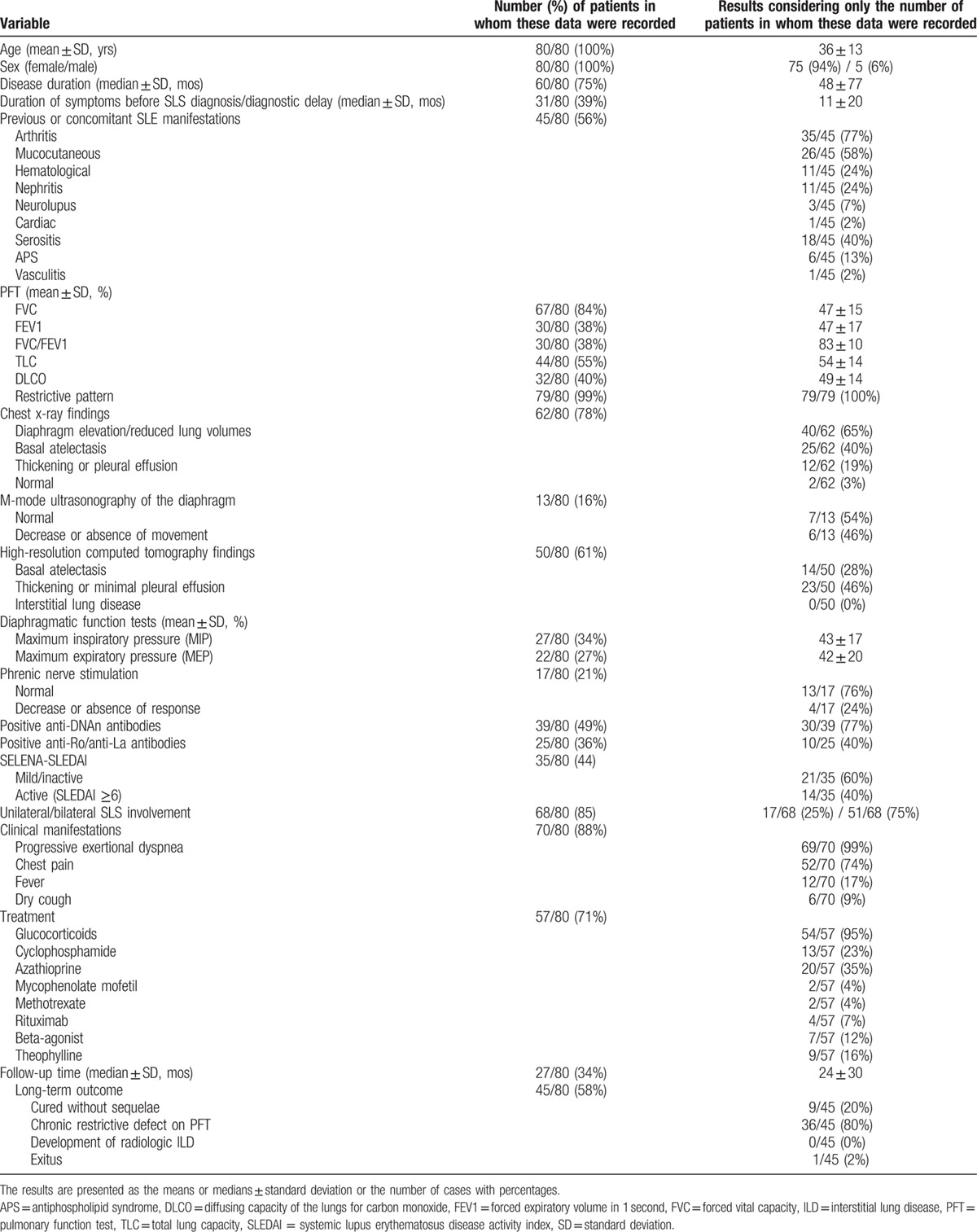
Clinical characteristics and outcome of 80 reported adult SLE patients with SLS.

The global analysis of the 89 patients available for review provided the following information.

### 3.1 Demographic data

Of the 89 patients, 84 were women (94%) and 5 (6%) were men, with a female-to-male ratio of 17:1, and ages ranging from 19 to 69 years (mean 36 ± 12 years). The median duration of SLE before the diagnosis of SLS was 4 ± 6.5 years (range 1–420 months). In 9% of the cases (6/69), SLS was the presenting feature of SLE.

### 3.2 Other previous and concomitant clinical manifestations of SLE

The majority of patients did not have previous or concomitant major organ involvement other than SLS. The most common clinical features of lupus were arthritis and dermatologic involvement and, less commonly, serositis (mainly pleuritis). Nephritis was documented in 31% (17/54) of the patients.

Regarding their autoantibody profile, 81% (39/48) of patients tested positive for anti-dsDNA antibodies, whereas anti-SSA/Ro antibodies were positive in only 35% (11/31) of the patients in whom this information was available.

Active disease (defined as a SELENA-SLEDAI score ≥6) at SLS diagnosis was present in 44% (18/41) of the patients. Only 15% (6/41) of them had a severe lupus flare (defined as a SELENA-SLEDAI score ≥12).

### 3.3 Presenting features of SLS

Almost all patients (88/89) presented exertional dyspnea of variable severity that progressed in some cases, over different periods, to breathlessness at rest. Concomitant pleuritic chest pain was common, being reported in 76% (60/79) of the patients. Orthopnea may also occur because of diaphragmatic weakness. Fever or dry cough was more rarely seen (19% [15/79] and 8% [6/79], respectively). In 73% (56/77) of patients, the syndrome was bilateral.

The most frequent findings on clinical examination were tachypnea associated with limited chest wall examination, frequently with use of the accessory muscles of respiration. Lung auscultation often is normal, although bibasal crepitations may be heard in the presence of basal atelectasis.

An important diagnostic delay was common. The mean duration of symptoms before the diagnosis was 11 ± 20 months. In 48% (15/31) of the patients, the average duration of symptoms before the SLS diagnosis was longer than a year.

### 3.4 Imaging techniques

In the majority of patients, both chest x-ray and thoracic HRCT was employed to establish the diagnosis. The most frequent chest x-ray finding was elevated unilateral or bilateral hemidiaphragms, with reduced lung volumes being observed in 69% of patients (49/71). In some, it was associated with basal linear atelectasis (41% [29/71]) and/or mild pleural effusions or pleural thickening (23% [16/71]). The chest radiograph was described as normal in only 3% of the patients (2/62).

High-resolution computed tomography was more sensitive than x-ray in detecting mild pleural effusions/thickening or atelectasis (47% [28/59] and 37% [22/59] of the cases, respectively), but its main utility was in ruling out the presence of parenchymal lung disease or vascular pathology.

The usefulness of the evaluation of diaphragm dome motion by M-mode ultrasonography was lesser, establishing the diagnosis only in 46% (6/13) of the patients. The paralyzed side shows no active caudal movement of the diaphragm, with inspiration and abnormal paradoxical movement (i.e., cranial movement on inspiration), particularly with the sniff test.

Dynamic contrast-enhanced lung magnetic resonance imaging (MRI) was employed only in 1 case.

### 3.5 Pulmonary and diaphragmatic function tests

Results of PFT were abnormal in all patients described, showing characteristically a restrictive defect with reduced lung volumes and carbon monoxide gas transfer capacity (DLCO).

Forced vital capacity (FVC) ranged from 21% to 80% of predicted capacity, with an overall mean value of 48 ± 16%. Both the total lung capacity (TLC) and the DLCO were reduced (mean values of 57 ± 17% and 48 ± 16% of predicted, respectively), whereas the carbon monoxide transfer coefficient (KCO) (i.e., corrected for alveolar volume) was generally normal.

Measures of diaphragmatic muscle strength were also of great utility to confirm the suspicion of SLS. The great majority of these patients (89% [32/36]) had a decrease in maximal inspiratory pressure (MIP) and maximal expiratory pressure (MEP), indicating a global respiratory muscle weakness. The overall mean values of MIP and MEP were reduced: 43 ± 17% and 42 ± 20% of the predicted values, respectively.

### 3.6 Electromyography

Phrenic nerve conduction studies were performed in 17 patients, and showed normal results in 13 (76%), and also decreased or absent response to stimulation in the remaining 4.

### 3.7 Treatment

The great majority of patients (95%) [63/66] were treated with medium or high doses of glucocorticoids (53% [35/66] were treated with initial doses >30 mg/daily of prednisone or equivalent). Two patients received intravenous methylprednisolone pulse therapy (1 g daily for 3 days), followed by oral corticosteroids.

In more than half of the patients, immunosuppressive agents were used in conjunction with steroids since the beginning of the treatment, or when the patient failed to improve. The most frequently used were azathioprine (20 cases) and cyclophosphamide (13 cases). There is also limited experience with mycophenolate mofetil and methotrexate. Rituximab (RTX) is increasingly being used in severe or refractory cases of SLS (9 patients), with effectiveness and good safety profile.^[22,52,54,59]^

Theophylline (15% [10/66] of cases) and beta-agonists (20% [13/66]), alone or in combination with glucocorticoids, have been also employed with the intent to increase diaphragmatic strength.

Information about the duration of applied treatment, particularly with respect to glucocorticoid regimen and tapering, was not included in the considered articles.

### 3.8 Outcome

Information about follow-up was available in only 62% (55/89) of patients. The median of follow-up period was 35 ± 29 months (range from 6 months to 9 years).

Of the 55 patients, 18% (10/55) cured without functional sequelae; the other patients had significant clinical improvement with stabilization or mild to moderate improvement on PFT after treatment. Despite the persistence of a chronic restrictive defect in these patients, none required chronic home oxygen therapy.

Only 1 patient died because of pneumonia,^[42]^ but she also had concomitant active lupus nephritis and poor medication adherence. In addition, 1 patient finally developed interstitial lung disease (nonspecific interstitial pneumonitis) 4 years after SLS onset.

## 4 Discussion

Shrinking lung syndrome is a rare complication of SLE. Our data confirm its low prevalence, estimated between 0.5% and 1.1% in the general lupus population.^[24,50]^ It may complicate SLE at any time over its course, ranging from as early as 1 month to 35 years after disease onset. Although it is much more frequent in advanced stages of the disease, rarely, SLS may be the presenting manifestation of SLE.^[43,48,57,66,69,76]^ This complication is more common in SLE patients of female sex, with a female-to-male ratio of 17:1.

The major symptom of SLS is progressive exertional dyspnea of variable severity, accompanied by pleuritic chest pain in three-quarters of patients. This complication usually occurs in patients without previous or concomitant major organ involvement other than SLS, and, in more than half of the patients, in inactive stages of the disease, according to SELENA-SLEDAI scores.^[43–77]^

An important diagnostic delay is observed in the clinical practice, indicating that SLS is still an under-recognized pulmonary complication of SLE. Despite its rarity, SLS should be considered in the differential diagnosis of lupus patients with dyspnea and/or pleuritic chest pain. In many ways, it represents a diagnosis of exclusion. In this sense, the recommended first-line procedures for diagnosis should include chest x-ray and thoracic HRCT, along with pulmonary and diaphragmatic function tests. The most distinctive findings of this entity were elevated unilateral or bilateral hemidiaphragms with reduced lung volumes, a restrictive ventilatory defect in PFT with reduced TLC and DLCO in absence of parenchymal lung disease or vascular pathology on imaging techniques, and reduced MIP and MEP.^[17,43–77]^

The evaluation of diaphragm dome motion by M-mode ultrasonography or dynamic contrast-enhanced lung MRI might be a useful second-line tool to reinforce clinical suspicion in cases of diagnostic difficulty. The accumulated experience with dynamic contrast-enhanced lung MRI is still scarce. In the only case reported to date,^[74]^ it detected the presence of subjacent pleural inflammation as an underlying cause of this condition, without evidence of myositic changes involving the diaphragm, and dynamic MRI sequences on forced voluntary ventilation confirmed physiologic movements of both diaphragms and all auxiliary respiratory muscles. According to the preliminary data, dynamic MRI might emerge as a useful second-line tool in the diagnosis of SLS. On the contrary, the diagnostic usefulness of the electromyography seems poor.^[16,17,45,66]^

There are no standardized guidelines for the treatment of SLS in SLE. According to the available experience, the majority of patients should be initially treated with medium or high doses of glucocorticoids.^[43–77]^ An immunosuppressive agent in conjunction with steroids should be used if the patient fails to improve, and it is advisable from the beginning of the treatment in patients with severe clinical and functional decline, but there are no randomized controlled trials to provide data concerning their efficacy. The most widely used drugs were azathioprine and cyclophosphamide, with variable success.^[43–77]^ Further, RTX is used in severe or refractory SLS (9 patients), with effectiveness and good safety profile in all cases.^[22,52,54,59]^ Based on this preliminary experience, RTX might emerge as the first-choice immunosuppressant, particularly in patients with severe or refractory disease, although larger-cohort studies must be performed in this field to confirm the efficacy of RTX in SLS. Theophylline and beta-agonists, alone or in combination with glucocorticoids, have also been suggested with the intent to increase diaphragmatic strength, but evidence of beneficial effects is lacking. Information about the duration of applied treatment, particularly with respect to glucocorticoid regimen and tapering, was not specified in the revised articles. In addition, the dose and duration of corticosteroids required for controlling SLS have never been tested in a randomized trial design. So it must be individualized based on the clinical response and evolution of pulmonary and diaphragmatic function tests, but taking also into account the recent recommendations for a more rational prescription of glucocorticoids.^[78,79]^

The overall response to treatment is positive. The great majority of patients had significant clinical improvement and stabilization, or mild to moderate improvement on PFT.^[17,43–77]^ Despite the persistence of a chronic restrictive defect in most of these patients, none required chronic home oxygen therapy, and death because of respiratory failure or associated complications was extremely rare, indicating a low-grade defect.

In interpreting the results of our study, one needs to consider its potential limitations derived from its observational nature, the small sample size, and the lack of multiple center's data. In addition, we cannot ignore the pitfalls inherent in any systematic review including the relatively small number of identified patients, the retrospective design, and incomplete follow-up data in some cases.

The precise pathogenic mechanism underlying the SLS remains controversial. Because pathologic data are unavailable, theories of its etiology are speculative. In the past, it has been attributed successively to a surfactant deficiency,^[15,43]^ isolated diaphragmatic myopathy,^[34,39,44,53,65,67]^ phrenic nerve dysfunction,^[45,66]^ chest wall dysfunction,^[19,71]^ and pleural adhesions,^[31,39]^ but none of these hypotheses has been unequivocally confirmed.

To date, data support the hypothesis that SLS may be an unusual complication of pleuritis, whereby inhibited respiratory muscle engagement limits inflation and thereby leads to progressive loss of lung compliance.^[16,48,51]^ Pleuritic chest pain is a prominent feature of patients with SLS. Our literature search revealed that 76% of SLS patients had active pleuritic chest pain at or shortly before clinical manifestation of the syndrome. The most likely cause of pain is inflammation of the pleural sheets, involving the diaphragmatic pleura or a pleural region near the diaphragm. The notion that pleurisy may be the underlying cause of the diaphragmatic dysfunction is further supported by the evidence of subjacent pleural inflammation, mild pleural effusions, or pleural thickening found in the imaging (chest x-ray, thoracic HRCT, and dynamic contrast-enhanced lung MRI) of about half of the SLS patients.

The mechanism by which pleural inflammation and pain may lead to diaphragm dysfunction is probably through reflex inhibition of diaphragmatic activation.^[16,48]^ Respiratory muscle function is regulated not only by volition but also through neuronal reflex arcs. These include the intercostal–phrenic and pleural–phrenic reflexes, which are activated by stimulation of intercostal and pleural afferents, respectively, leading to phrenic nerve inhibition.^[80–86]^ These reflexes have been documented in humans and may be responsible for decreased inspiratory capacity in patients after surgery.^[87]^ The pleural–phrenic reflex can be activated by exposure of the pleura to inflammatory cytokines.^[86]^ Therefore, it is likely that inflammation of the parietal pleura may lead to stimulation of unmyelinated or thin myelinated fibers belonging to the internal intercostal nerves that normally innervate the costal and the peripheral part of the diaphragmatic pleura.^[48]^ Pain that typically accompanies pleural inflammation has profound effects on pulmonary function. The localized guarding of the muscles in the area of pain, and also the generalized muscle rigidity, in association with a submaximal voluntary effort, lead to adoption of a swallow and rapid breathing pattern, which results in decreased functional residual capacity (FVC), forced expiratory volume in 1 second, and TLC.^[88]^

Therefore, it is hypothesized that pleural inflammation and pain trigger inhibition of deep inspiration by neural reflexes, resulting in chronic lung hypoinflation and decreased lung compliance.^[16,48]^ In this sense, a comparable decrease in lung compliance has been observed in patients who are chronically restricted to lower lung volumes, for example, patients with spinal cord injury or muscular dystrophy.^[89–92]^ The basis of the impairment of lung compliance is uncertain, although both a tissue remodeling with changes in the elasticity of the lung tissue and alterations in surfactant have been implicated as the underlying pathophysiology.^[16,93]^ Impaired compliance worsens hypoinflation, initiating a positive feedback loop that helps to explain the gradual progression of SLS. Because this defect is primarily functional, the patient's ventilatory drive would be expected to limit further respiratory deterioration, thus accounting for the low mortality of SLS despite its alarming clinical presentation. However, that nearly 25% of the reported SLS cases are not accompanied by pleuritic chest pain raises suspicion that the pathophysiology of SLS in this subgroup of patients may be related to another immunological cause that is responsive to anti-inflammatory and immunosuppressive agents.

In conclusion, SLS represents a rare complication of SLE. Although uncommon, it is important to be aware of its presenting features and prognosis because if it is not treated promptly and aggressively, it can lead to chronic restrictive ventilatory dysfunction. Further multicenter controlled studies are required to define the best diagnostic and treatment options for this complication.
